# Transaxillary Stenting of LVAD Outflow Graft Obstruction

**DOI:** 10.1016/j.jaccas.2021.02.038

**Published:** 2021-05-12

**Authors:** Erin Dreessens, Justin Cox, Jack Ramsey, Ensley Uyeda, Robert Adamson, Brian Jaski, Arvin Narula

**Affiliations:** a Naval Medical Center San Diego, San Diego, California, USA; b Sharp Memorial Hospital, San Diego, California, USA

**Keywords:** advanced heart failure, computed tomographic angiography, IVUS, left ventricular assist device, LVAD stenting, percutaneous intervention, INR, international normalized ratio, IVUS, intravascular ultrasound, LVAD, left ventricular assist device, OGO, outflow graft obstruction, PTFE, polytetrafluoroethylene

## Abstract

An 82-year-old man with a HeartMate II left ventricular assist device presented with low-flow alarms and cardiogenic shock secondary to left ventricular assist device outflow graft obstruction. Given high risk for redo sternotomy, the heart team decided on percutaneous intervention with peripheral stents, a procedure that is currently limited to case reports. (**Level of Difficulty: Advanced.**)

## Central Illustration

**Figure:**
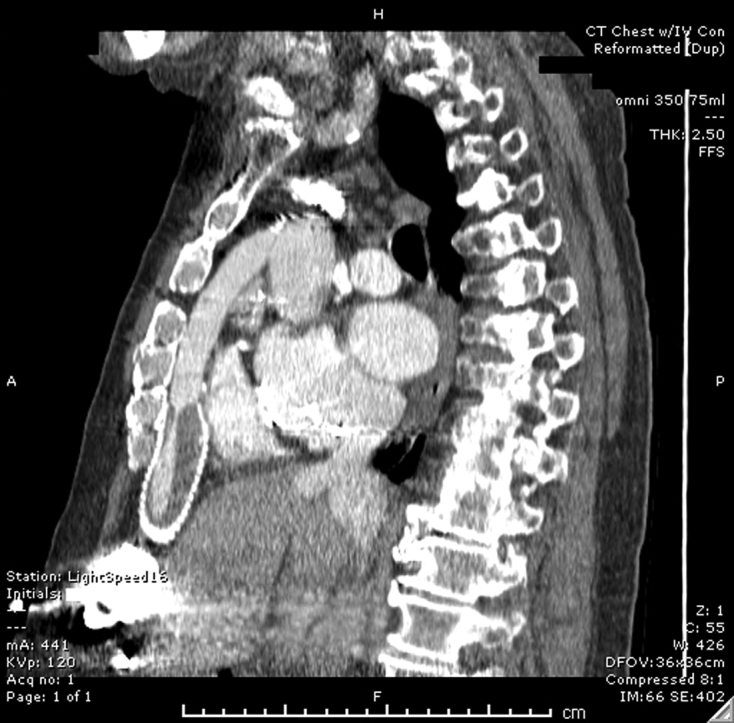

## History of Presentation

An 82-year-old man with ischemic cardiomyopathy status post HeartMate II (Abbott Cardiovascular, Plymouth, Minnesota) left ventricular assist device (LVAD) implantation in 2013 presented in cardiogenic shock with low-flow alarms. Admission vital signs were a pulse of 100 beats/min, blood pressure 84 mm Hg by Doppler, respiratory rate 22 breaths/min, 90% oxygen saturation on room air, and 22-lb weight gain. Physical examination was significant for LVAD hum, bibasilar crackles, and 2+ pitting edema of the lower extremities. The 24-h LVAD readings showed pump speed of 9200 RPM, flow of 3.6 l/min (range: 2.6 to 5), PI 5.0, and power 5.0 W. During his stay, alarm frequency increased to 42 red heart alarms the night before intervention.

**Learning Objectives**

• To be able to make a differential diagnosis of LVAD OGO using multimodality imaging.
• To obtain IVUS images in patients with LVAD OGO to determine etiology and assist with perioperative planning for stroke prevention.
• To consider use of the axillary artery for vascular access if stent delivery catheter lengths preclude femoral access.

## Medical History

Additional medical history included atrial fibrillation and dual-chamber pacemaker implantation. Home medications included warfarin, aspirin, sildenafil, hydralazine, digoxin, and amiodarone. Diuretic agents had been discontinued 1 month previously because of recurrent pre-syncope.

## Differential Diagnosis

The differential diagnosis for low-flow alarms includes hypovolemia, acute blood loss, right-sided heart failure, inflow graft obstruction, or outflow graft obstruction (OGO) (1). OGOs are further subdivided into thrombosis (34.6%), stenosis (34.6%), kinking (11.5%), pseudoaneurysm (3.8%), external graft compression (3.8%), and bronchial artery fistula (3.8%) (2).

## Investigations

**Figure 1:**
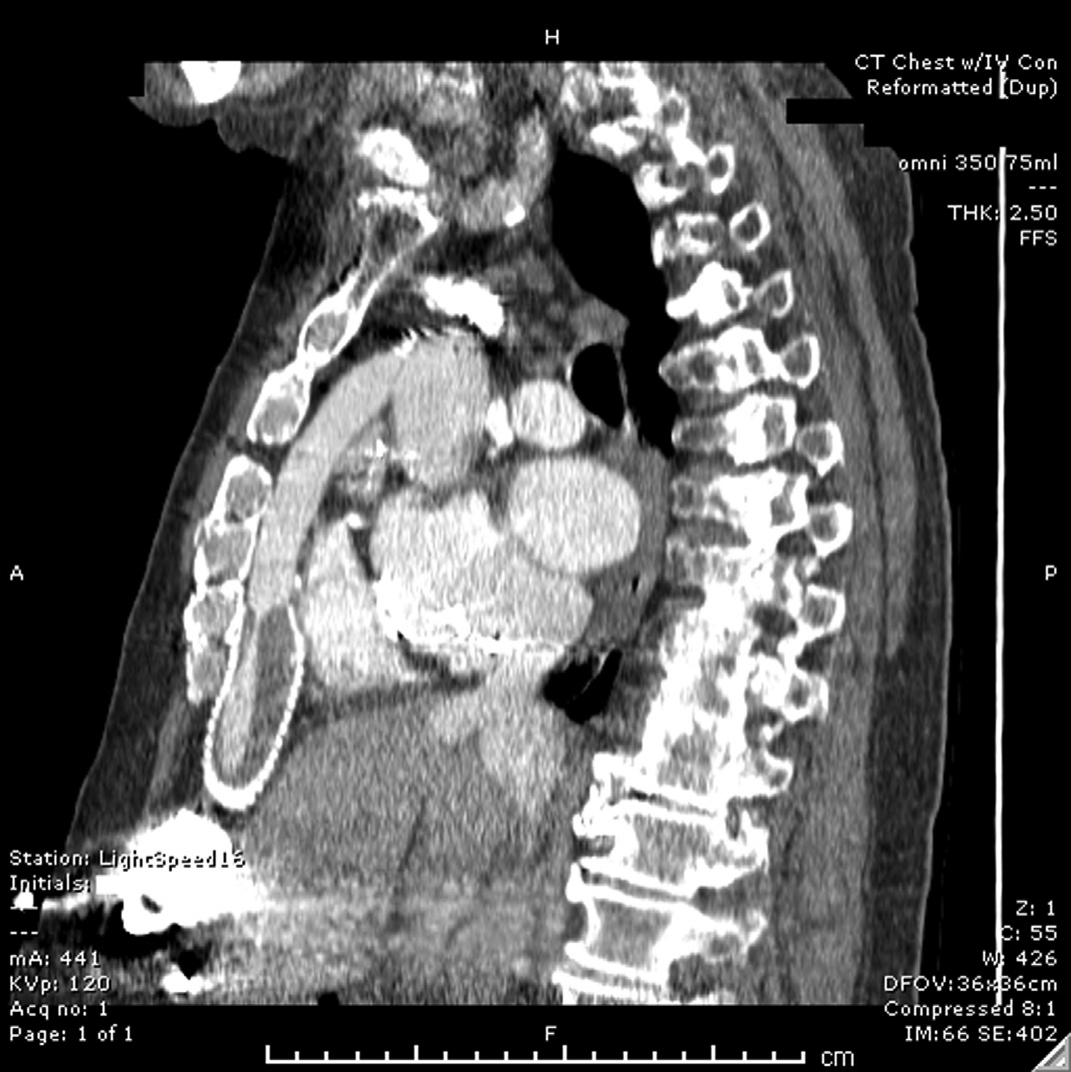
Sagittal Computed Tomography Demonstrating Outflow Graft Obstruction

**Figure 2:**
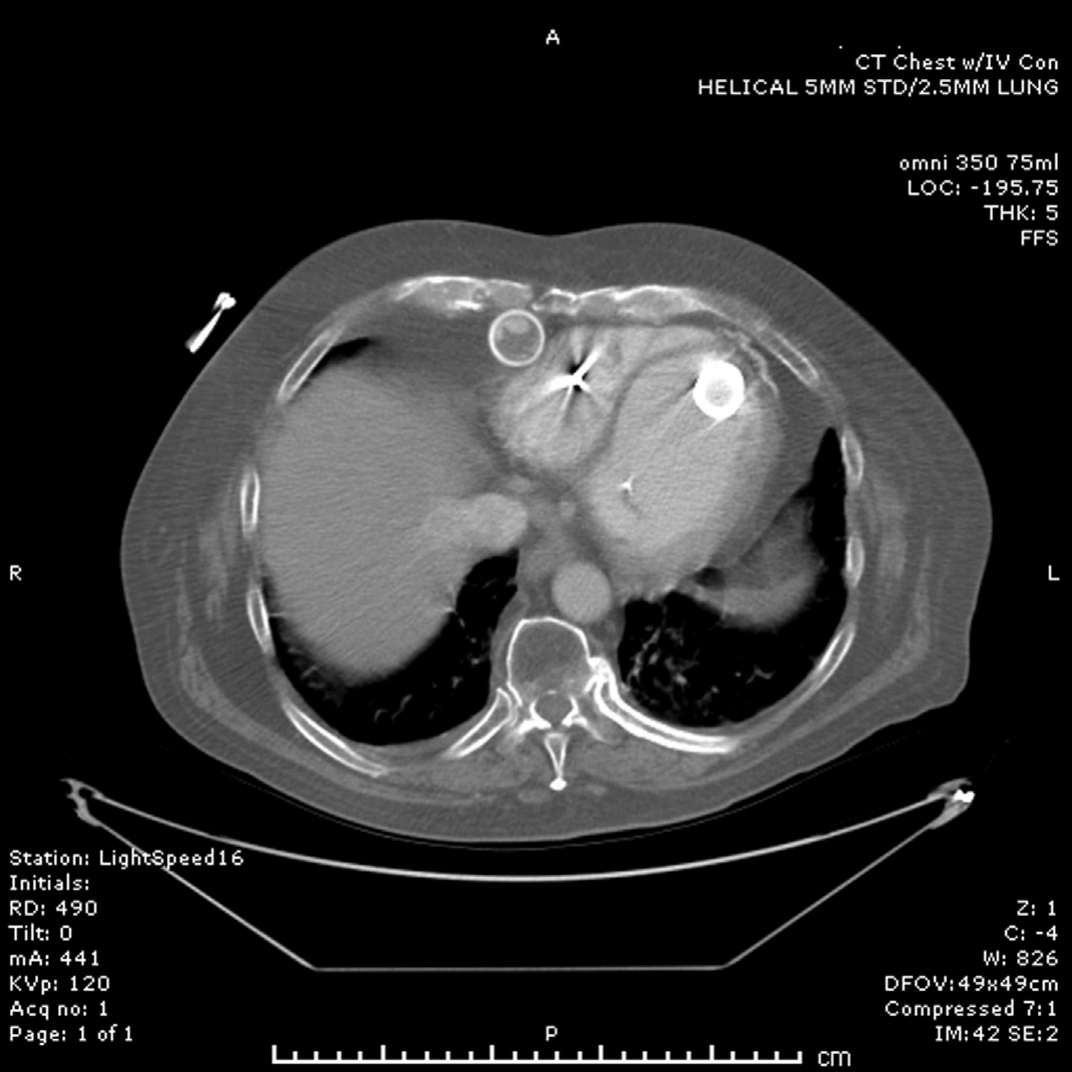
Axial Computed Tomography Demonstrating Outflow Graft Obstruction

Admission laboratory assessments showed a therapeutic international normalized ratio (INR) (2.8), stable creatinine (1.2 mg/dL), stable anemia (hemoglobin 9.6 g/dL), and mildly elevated lactate dehydrogenase (390 U/l; upper limit of normal 225 U/l). Echocardiography showed severe left ventricular dilation, increased from 6 months previously. Right heart catheterization showed a cardiac index of 1.54 l/min/m^2^ and mixed venous saturation of 44%. Computed tomography confirmed OGO (Figures 1 and 2).

## Management

**Figure 3:**
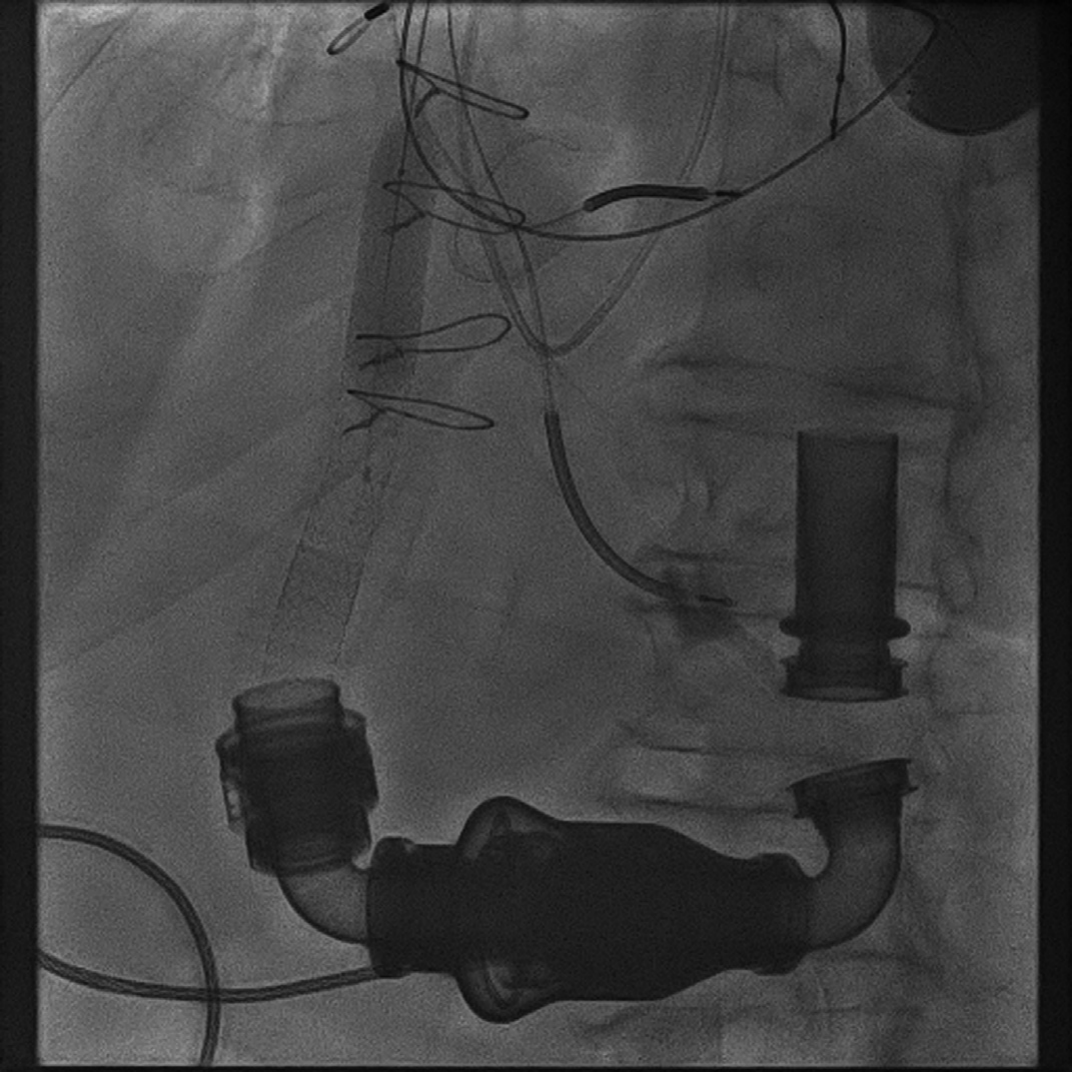
Representative Image of Post-Dilation of Stent in the Outflow Graft

**Figure 4:**
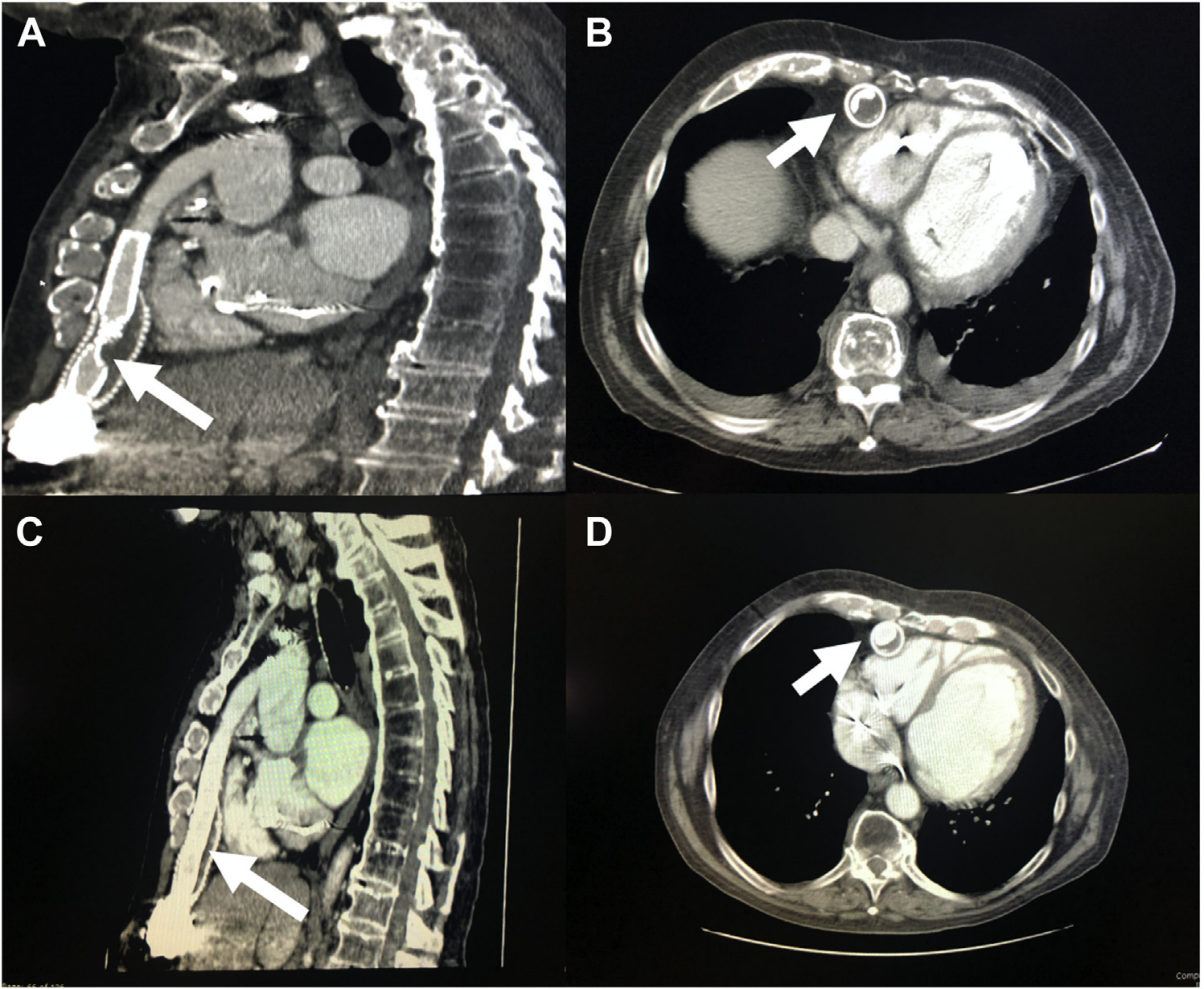
Representative Computed Tomographic Images Demonstrating Kinked Viabahn Stent and Fully Deployed Vici Stent **(A, B)** Kinked Viabahn stent. **(C, D)** Fully deployed Vici stent.

The patient was presented to our multidisciplinary heart team and believed to be high risk for repeat surgery. Warfarin was held leading up to the procedure, and the INR was 1.7 on the morning of the procedure. We pre-emptively elected to use a Sentinel protection device (Boston Scientific, Marlborough, Massachusetts) via right radial access and Viabahn covered stents (Gore Medical, Newark, Delaware) in our initial procedure because our patient’s LVAD OGO embolization risk was uncertain. Transaxillary access was chosen because of the use of short-shaft balloons, which would not reach the outflow graft via femoral access, and the need for 8-F access, which would preclude a radial approach. Using a micropuncture needle under ultrasound guidance, the left axillary artery was cannulated and an 8-F Pinnacle sheath (Terumo, Tokyo, Japan) was placed after pre-closing the artery. Next, the obstruction was evaluated on intravascular ultrasound (IVUS), which demonstrated material between the graft and outflow sleeve causing obstruction. The minimal luminal area measured 44.6 mm^2^ (Video 1). A Viabahn VBX 11 × 79 mm stent was deployed at nominal pressure, while LVAD flow was turned down to 6,000 rpm to prevent stent displacement. The stent was post-dilated with a 14 × 60 mm noncompliant Atlas balloon (Becton Dickinson, Franklin Lakes, New Jersey). Repeat IVUS revealed that the compressive material tended to move superior and inferior to stent placement (Video 2), necessitating stenting of the entire outflow graft with a Viabahn VBX 11 × 59 mm stent inferiorly and a Viabahn VBX 11 × 59 mm superiorly (Figure 3). Final IVUS showed a minimal luminal area of 106 mm^2^, and LVAD outflow improved from 2.5 to 5 l/min. After cinching the Perclose (Abbott Cardiovascular), final access-site angiography showed no evidence of bleeding or aneurysm (Video 3). Unfortunately, the patient developed a late hematoma that night requiring 3 U packed red blood cells. The INR the following morning was 1.6, and warfarin was restarted. He was discharged to home 3 days after the procedure with no further bleeding or alarms. He returned 2 months later with low-flow alarms, and computed tomography showed a new OGO, this time with a kink in the stent column. He was again stented, this time with a Vici 16 × 90 cm expandable stent (Boston Scientific), with the hope that higher radial force would limit rebound compression (Figure 4).

## Discussion

OGO should be suspected if LVAD low-flow alarms occur associated with outflow graft velocity change, left ventricular dilation, or worsening mitral regurgitation. Proposed mechanisms for nonthrombus stenosis include neointimal hyperplasia, kinking of the sleeve, or blood-derived proteinaceous materials between the graft and outflow sleeve causing compression, which surgeons have observed on LVAD explants (3).

In particular, proteinaceous material has been observed between the outflow graft and bend relief in most cases (4). It is believed that the outflow graft allows seeping of blood and protein through the graft material, similar to a type IV endograft leak. The outflow graft covering, typically composed of polytetrafluoroethylene (PTFE), has different porosity and in some patients does not allow absorption of these materials. Thus, it becomes trapped between the outflow graft and PTFE covering, eventually compressing the outflow graft. This phenomenon can occur despite a therapeutic INR. PTFE coverings are used in HeartMate II and HeartWare (Medtronic, Minneapolis, Minnesota) LVADs (5). OGO from this mechanism is also reported with the HeartMate III (6).

Stenting of OGO is the preferred treatment, followed by corrective surgery if unsuccessful (7). A systematic review of limited available case reports published in 2019 showed 88.5% immediate success of stenting, with acceptable 30-day mortality of 6.7% and overall mortality of 9.5% (2). Both femoral and transaxillary approaches have been described (8).

Covered stents are necessary if there is thrombus or material within the outflow graft at risk for embolization (7). However, if the material is between the graft and outflow sleeve, the risk for embolism is reduced. We found IVUS to be useful for determining the cause of obstruction and believe that it should be incorporated into the diagnostic algorithm for OGO. If IVUS interrogation reveals external compression from proteinaceous material, it would obviate the need for sentinel protection or covered stents, decreasing an access point and lowering bleed risk.

IVUS also helped identify propagation of the material intraprocedurally, which ultimately led to stenting of the entire outflow graft. On this basis, we believe the longest stent possible should be used to limit propagation of the obstruction, particularly during post-dilation, and IVUS should be performed to verify that all material is covered.

Finally, case reports have shown recurrent obstruction, with 2 prior reported incidents 76 and 556 days post-stenting (3). To our knowledge, this is the first report of a kinked stent within an outflow graft. We postulate that stents with higher radial force, such as Vici or Palmaz-Schatz stents, may be a superior choice to prevent this phenomenon. On the basis of limited case series and our experience, we also believe that interval follow-up imaging should be considered in these patients to evaluate for repeat obstruction.

## Follow-Up

Our patient remains well since his second procedure and has had no further alarms. Follow-up imaging 3.5 months later confirmed that no further OGO had occurred.

## Conclusions

LVADs are used with increased frequency in patients with advanced heart failure. Understanding device complications and management options will be vital to their long-term management. We present our experiences in percutaneous intervention on LVAD OGO and key points we learned that may assist others in optimizing their results with this procedure.

## Funding Support and Author Disclosures

The authors have reported that they have no relationships relevant to the contents of this paper to disclose.

## Appendix

Supplemental Video 1 Initial Intravascular Ultrasound Showing Outflow Graft Obstruction

Supplemental Video 2 Intravascular Ultrasound After Stent Deployment Showing Superior Propagation of Proteinaceous Materials

Supplemental Video 3 Final Axillary Access Shot Confirming no Bleeding or Aneurysm
